# Removal of dental implant displaced into maxillary sinus by combination of endoscopically assisted and bone repositioning techniques: a case report

**DOI:** 10.1186/s13256-015-0787-1

**Published:** 2016-01-12

**Authors:** Shinnosuke Nogami, Kensuke Yamauchi, Yuji Tanuma, Kenji Odashima, Aritsune Matsui, Kenko Tanaka, Tetsu Takahashi

**Affiliations:** Division of Oral and Maxillofacial Surgery, Department of Oral Medicine and Surgery, Tohoku University Graduate School of Dentistry, 4-1 Seiryo-machi, Aoba-ku, Sendai, 980-8575 Miyagi Japan

**Keywords:** Endoscopic surgery, Implant displacement, Maxillary sinus, Migrated implants

## Abstract

**Background:**

Accidental displacement of a dental implant into the maxillary sinus is an infrequent although not uncommon complication encountered in dental clinical practice, with the main cause thought to be inadequate bone height in the posterior maxilla. We report a case of migration of a dental implant into the maxillary sinus, and discuss the benefits of its removal by a combination of endoscopically assisted and bone repositioning techniques.

**Case presentation:**

A 35-year-old Japanese man with a partially edentulous maxilla underwent implant placement at a private clinic. Three months later, at the time of abutment connection, the implant at the site of his maxillary right first molar was accidentally pushed into the sinus. The hole on the alveolar ridge made for placement of the implant was small and far from the dislocated implant, thus access was achieved in a transoral manner via the frontal wall of his maxillary sinus with an endoscopic approach. Piezoelectric instruments were used to perform an osteotomy. The bone lid was removed, and the implant was identified using a rigid endoscope and removed with a surgical aspirator, followed by repositioning of the bony segment; the area was secured with an absorbable suture. Removal of migrated implants should be considered in order to prevent possible sinusal disease complications.

**Conclusions:**

In the present case, removal of a dental implant displaced into the maxillary sinus by use of a combination of endoscopically assisted and bone repositioning techniques proved to be a safe and reliable procedure.

## Background

Rehabilitation of an edentulous jaw with an implant-supported prosthesis has become common practice among oral surgeons and dentists over the past 3 decades [1]. However, reabsorption of the alveolar ridge in the posterior maxilla and/or maxillary sinus pneumatization often limits the amount of available bone for positioning dental implants. To overcome these problems, use of short implants or maxillary sinus floor lifting in association with dental implants is well documented and has proven to be successful [2, 3].

Accidental displacement of roots, endodontic materials, and dental implants into the maxillary sinus is a relatively common complication encountered in dental clinical practice [4], although dental implant displacement can cause serious complications [5]. The aim of this report is to present an unusual clinical case of migration of a displaced implant in the maxilla towards the anterior of the maxillary sinus and its subsequent removal.

## Case presentation

**Fig. 1:**
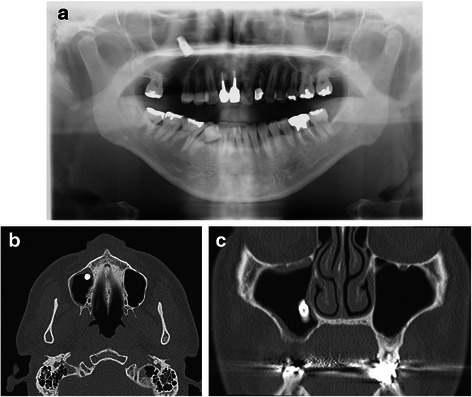
**a** Panoramic radiograph. **b, c** Computed tomography images obtained prior to the operation (**b**, axial section; **c**, coronal section)

A 35-year-old Japanese man with a partially edentulous maxilla underwent implant placement at a private clinic. Three months after that procedure, at the time of abutment connection, the implant at the site of his maxillary right first molar was accidentally pushed into the sinus. The attending dentist did not immediately remove the dental fixture and referred the patient to our department at 16 months after initial placement of the affected implant. At the initial examination, we noted that the patient had slight pain in his right posterior maxilla. Panoramic radiograph and computed tomography (CT) images showed what appeared to be the implant apparently dislocated to within the anterior part of his maxillary sinus (Fig. 1a, b, c). Because the hole on the alveolar ridge that was made for placement was small and far from the dislocated implant, access was achieved in a transoral manner via the frontal wall of his maxillary sinus using an endoscopic approach.

**Fig. 2:**
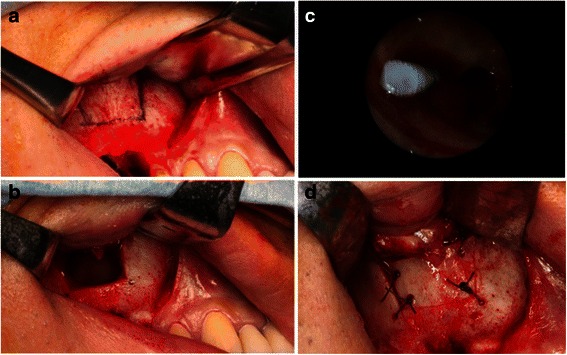
**a** Osteotomy line of bony window. **b** The bone lid was removed. **c** Endoscopic view of migrated dental implant in the right maxillary sinus. **d** The bony segment was repositioned and secured with an absorbable suture

**Fig. 3:**
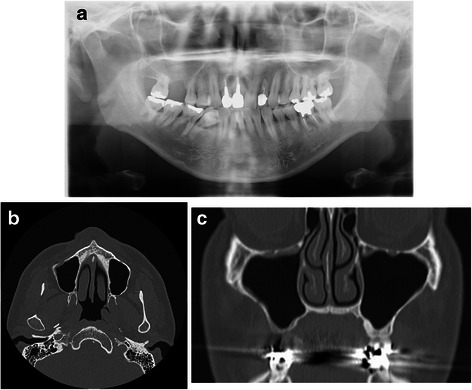
**a** Panoramic radiograph obtained 1 week after surgery. **b, c** Computed tomography images obtained 6 months after surgery (**b**, axial section; **c**, coronal section)

Under local anesthesia, an intraoral approach consisting of elevation of the mucoperiosteal flap and creation of a bony window pedicled to the Schneiderian membrane was utilized. Surgical intervention began with elevation of a trapezoidal full-thickness mucoperiosteal flap (Fig. 2a); then a low-speed straight handpiece and fissure bur were used to drill four holes in his maxillary bone. Piezoelectric instruments were used to perform an osteotomy. The bone lid was removed (Fig. 2b), and we inserted a rigid endoscope 4 mm in diameter equipped with a digital video unit (KARL STORZ Endoscopy Japan Inc., Tokyo, Japan) into the created bony window. The implant was identified using a rigid endoscope and removed with a surgical aspirator (Fig. 2c). The bony segment was then repositioned and secured with an absorbable suture (Fig. 2d). After 7 days, the sutures were removed and a follow-up examination was performed, including panoramic X-ray imaging (Fig. 3a). At 6 months after removal of the displaced implant CT images were obtained (Fig. 3b, c). The patient provided informed consent regarding the treatment plan and procedure.

## Discussion

Implants placed in the posterior maxilla sometimes fail because of the presence of thin cortical bone with very low density in that area. The location of the maxillary sinus can also lead to unique complications, including maxillary sinusitis, an oroantral fistula, and implant displacement [6], with reports of displacement of dental implants into the maxillary sinus increasing [7]. One such report described delayed removal of a displaced implant that had relocated to the sphenoid sinus from the antrum [5], while another noted displaced implant migration after a period of 8 years [8]. In the present case, panoramic radiography performed 3 months after replacement implant surgery showed displacement of the implant into the maxillary sinus. Furthermore, slight sinusitis was revealed by CT and the patient had minor pain in the right posterior maxilla. Therefore, we soon performed surgery to remove the displaced implant from its point of migration in his maxillary sinus.

In general, two main treatment modalities have been proposed for removal of a displaced implant from the sinus and to treat associated infectious complications; an intraoral approach with creation of a window in the anterior-lateral wall of the maxillary sinus and a transnasal approach for functional endoscopic sinus surgery [5, 9]. A previous study investigated complications associated with our alternative method and compared them to those seen with the classic Caldwell–Luc procedure [10]. The present procedure has an advantage of good visualization, whereas use of a sublabial incision results in significant postoperative discomfort. Furthermore, subperiosteal dissection can injure branches of the infraorbital nerve, with subsequent anesthesia of the gingiva-buccal mucosa and teeth, while the Caldwell–Luc approach has an attendant risk of causing an oroantral fistula if the periosteum is not adequately closed.

Minimally invasive endoscopic surgery has been proposed for various indications in the craniomaxillofacial area. To minimize complications such as nerve damage and visible scars following wide skin incisions, endoscopic and endoscopy-assisted surgical procedures that utilize limited incisions have been developed in the past decade [11]. Although dependent on the presence of disease in the maxillary sinus, Nakamura *et al*. reported that endoscopic removal of a dental implant displaced into the maxillary sinus was useful [12]. However, contrasting findings indicate that endoscopic surgery is of limited value when treating odontogenic lesions or removing teeth, implants, or foreign bodies [13].

In the present case, we removed a migrated implant by use of a combination of endoscopically assisted and bone repositioning techniques. An osteotomy for bony window creation can be performed with rotary instruments or piezoelectric instrumentation, with the former widely used and well documented to provide a fast and effective osteotomy path. Also, recently introduced piezoelectric instruments utilize micro-vibration of surgical inserts at ultrasonic frequencies to perform cutting of hard tissues, and have been demonstrated to offer good cutting properties with cortical bone and preserve soft tissues from damage in cases of accidental contact [14]. Endoscopic removal is a minimally invasive procedure with reduced operative time and postoperative recovery, while it also provides superior visibility with a limited incision and respects the integrity of the sinus. In the present case, we were able to manipulate the endoscope tip to a position immediately facing the implant without trouble and removal from the access hole was easily achieved. Moreover, endoscopy is safe and does not damage soft tissues including the sinus mucosa. We confirmed that the procedure used in the present case did not damage soft tissues and resulted in decreased hypertrophy of the mucous membrane of the maxillary sinus at 6 months after removal surgery. Nevertheless, continued follow-up examinations are needed.

## Conclusions

In the present case, removal of a dental implant displaced into the maxillary sinus by use of a combination of endoscopically assisted and bone repositioning techniques proved to be a safe and reliable procedure.

## Consent

Written informed consent was obtained from the patient for publication of this case report and any accompanying images. A copy of the written consent is available for review by contacting the Editor-in-Chief of the journal.

## Abbreviation

CT: computed tomography
